# Novel and de novo mutation of *PCDH19* in Girls Clustering Epilepsy

**DOI:** 10.1002/brb3.1455

**Published:** 2019-11-12

**Authors:** Li Yang, Jing Liu, Quanping Su, Yufen Li, Xiaofan Yang, Liyun Xu, Lili Tong, Baomin Li

**Affiliations:** ^1^ Department of Pediatrics Qilu Hospital Affiliated to Shandong University Jinan China; ^2^ Department of Pediatrics Linyi People's Hospital Affiliated to Shandong University Linyi China; ^3^ Central Laboratory Linyi People's Hospital Affiliated to Shandong University Linyi China

**Keywords:** epilepsy, females, fever, later‐onset, *PCDH19*

## Abstract

**Background:**

*PCDH19* has become the second most relevant gene in epilepsy after *SCN1A*. Seizures often provoked by fever.

**Methods:**

We screened 152 children with fever‐sensitive epilepsy for gene detection. Their clinical information was followed up.

**Results:**

We found eight *PCDH19* point mutations (four novel and four reported) and one whole gene deletion in 10 female probands (seven sporadic cases and three family cases) who also had cluster seizures. The common clinical features of 16 patients in 10 families included fever‐sensitive and cluster seizures, mainly focal or tonic‐clonic seizures, and absence of status epilepticus, normal intelligence, or mild‐to‐moderate cognitive impairment, the onset age ranges from 5 months to 20 years. Only four patients had multiple or focal transient discharges in interictal EEG. Focal seizures originating in the frontal region were recorded in four patients, two from the parietal region, and one from the occipital region.

**Conclusion:**

*PCDH19* mutation can be inherited or de novo. The clinical spectrum of *PCDH19* mutation includes *PCDH19* Girls Clustering Epilepsy with or without mental retardation, psychosis, and asymptomatic male. The onset age of *PCDH19* Girls Clustering Epilepsy can range from infancy to adulthood. Sisters in the same family may be sensitive to the same antiepileptic drugs. And our report expands the mutation spectrum of *PCDH19* Girls Clustering Epilepsy.

## Graphical abstract

The onset age of PCDH19 Girl Clustering Epilepsy can range from infancy to adulthood. There was little difference in clinical phenotype and drug efficacy between sisters. And our report expands the mutation spectrum of PCDH19 Girl Clustering Epilepsy.

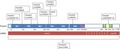

## 1 INTRODUCTION

Now, *PCDH19* has become the second most relevant gene in epilepsy after *SCN1A* (Depienne & LeGuern, 2012; Duszyc, Terczynska, & Hoffman‐Zacharska, 2015). *PCDH19* is located on chromosome X and is composed of six exons. *PCDH19‐*related epilepsy is characterized by incomplete penetration rate and phenotypic heterogeneity. Phenotypes ranged from mild epilepsy to epileptic encephalopathy, with epilepsy and mental retardation limited to females (EFMR), Dravet syndrome (DS), and genetic epilepsy with febrile seizures plus (GEFS+) (Specchio et al., 2011). EFMR as the main clinical phenotype is characterized by seizures with an early seizure onset and cognitive impairment. Seizures often occur in clusters and are often provoked by fever. With the expansion of phenotypic spectrum in *PCDH19* mutation patients, some patients do not have severe intellectual disability. Subsequent studies highlighted that most patients had focal epilepsy clusters triggered by fever, so “*PCDH19* girls clustering epilepsy” (Homan et al., 2018; Vlaskamp et al., 2019) (*PCDH19*‐GCE) was proposed as a name to facilitate clinical identification of this disorder.

The reported *PCDH19* mutations were mostly located at exon 1 (Depienne et al., 2011; Leonardi et al., 2014), which encodes the entire extracellular domain. About one‐half of the reported mutations leading to *PCDH19‐*related diseases are nonsense, frameshift, and splicing mutations, which severely truncate the protein protocadherin 19 (PCDH19). The remaining are missense mutations, and the missense mutations are concentrated in the extracellular domain of the protein (Kolc et al., 2019). The extracellular domain is essential for the normal function of the original cadherin function (Gerosa, Francolini, Bassani, & Passafaro, 2019). PCDH19 is mainly expressed in nerve tissues at different developmental stages, but its specific function is still unclear. Studies have shown that the function of PCDH19 may be related to neuronal connections and signal transduction on synaptic membranes (Duszyc et al., 2015). *PCDH19* mutation may lead to protein dysfunction.

In this study, we have screened 152 children with fever‐sensitive epilepsy and found *PCDH19* mutation in 10 female probands who also had cluster seizures with or without cognitive impairment or mental retardation in order to further understand the clinical and mutational features of *PCDH19*‐GCE.

## 2 METHODS

### 2.1 Subjects

We analyzed fever‐sensitive epilepsy children with the onset age 0–14 years between 2015 and 2019 in the Pediatrics Department of Qilu Hospital Affiliated to Shandong University and Linyi people's Hospital Affiliated to Shandong University, China. Exclusion criteria included seizures caused by nongenetic factors, such as an acquired brain injury (including traumatic brain injury, encephalitis, vasculitis, hypoxia, tumors, metabolic disorders, and toxicity); blood and urine screening indicate a metabolic disease; chromosome disease and clinically phenotypically defined monogenic diseases (e.g., tuberous sclerosis complex). Their clinical information was retrospectively collected and followed up, such as seizure types, onset age, treatment process, growth and development history, previous disease history, family history, degree of intellectual regression, physical examinations, autistic disorder test, cranial magnetic resonance imaging (MRI), and video‐EEG characteristics. The patients were followed up by phone or visit the clinic every 3 months. The study protocol was approved by the ethical committee of the Qilu Hospital Affiliated to Shandong University (No. 2016(027)) and Linyi People's Hospital Affiliated to Shandong University (No. 13003). All guardians signed informed consent forms.

### 2.2 Next‐generation sequencing (NGS) and DNA sequence analysis

Informed consent and blood samples were obtained from all the participants in the families. Genomic DNA was extracted using the QIAamp DNA Blood Mini Kit (Qiagen), according to the manufacturer's protocol. Each DNA sample is quantified by agarose gel electrophoresis and Nanodrop 2000 (Thermo). Libraries were prepared using Illumina standard protocol. The amplified DNA was captured with whole‐exon sequencing which contained the exons of *PCDH19* gene and its flanking UTRs. The capture experiment was conducted according to manufacturer's protocol. The junction sequences were trimmed, and the contamination or low‐quality reads were filtered for the raw data. Then, the clean data were aligned to the human reference genome sequence (hg19) by Burrows–Wheeler Alignment. Single‐nucleotide variation (SNV) and insertion–deletion mutation (InDel) were called by Genome Analysis Toolkit. Then all SNVs and InDels were annotated by ANNOVAR (RRID: SCR_012821)）. The mutation sites with frequencies <0.05 in the normal population database were screened out, including the 1,000 genome project, Exome Variant Server, and Exome Aggregation Consortium. Mutations were predicted by MutationTaster (MT), Sorting Intolerant From Tolerant (SIFT, RRID: SCR_012813), PolyPhen‐2 (PP2, RRID: SCR_013189), Genomic Evolutionary Rate Profiling (GERP++, RRID: SCR_000563), and Clustal‐W (RRID: SCR_017277). The selected mutation sites were verified by Sanger sequencing. The analysis of deletions or duplications was performed using multiplex ligation‐dependent probe amplification (MLPA) in those patients determined to be *PCDH19* mutation‐negative by Sanger sequencing.

## 3 RESULTS

### 3.1 Genetic analyses

**Figure 1:**
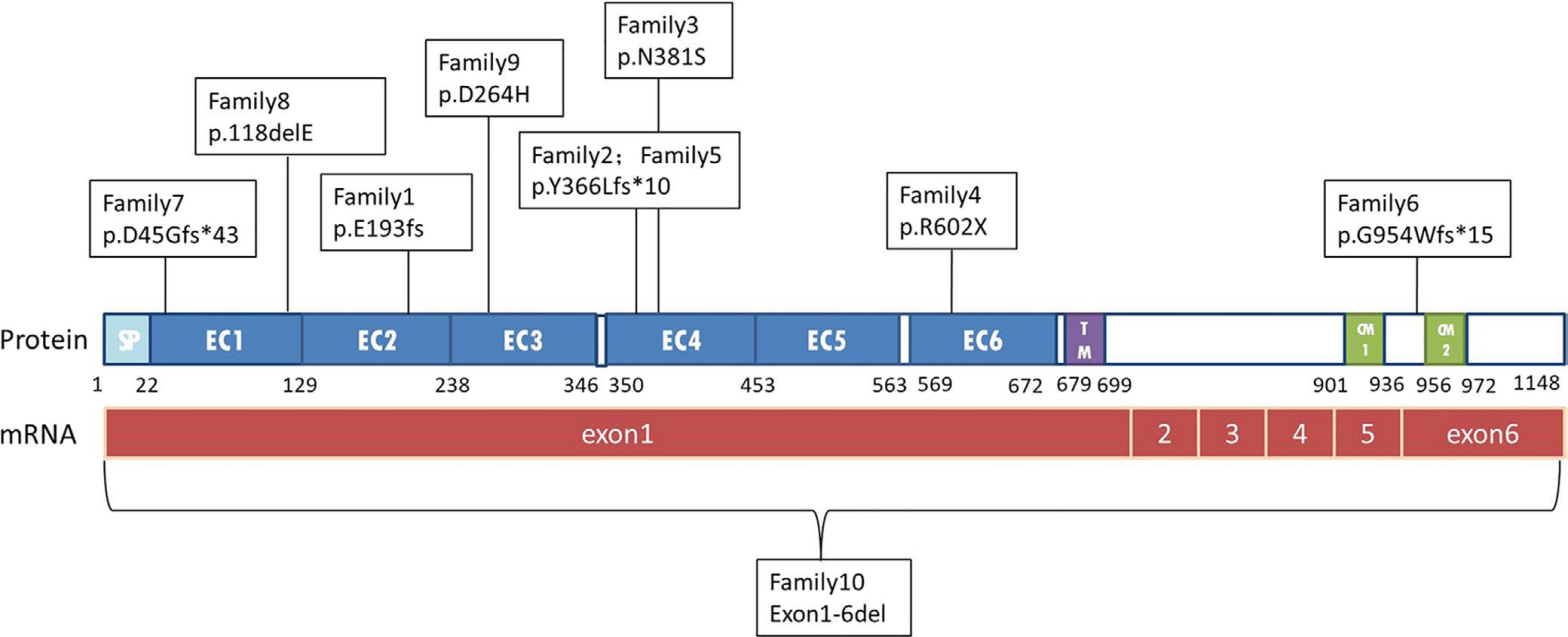
Schematic diagram of the mutations identified in the PCDH19 gene. CM1 and CM2, cytoplasmic domains 1 and 2; EC, extracellular cadherin domain; SP, signal peptide; TM, transmembrane domain

**Figure 2:**
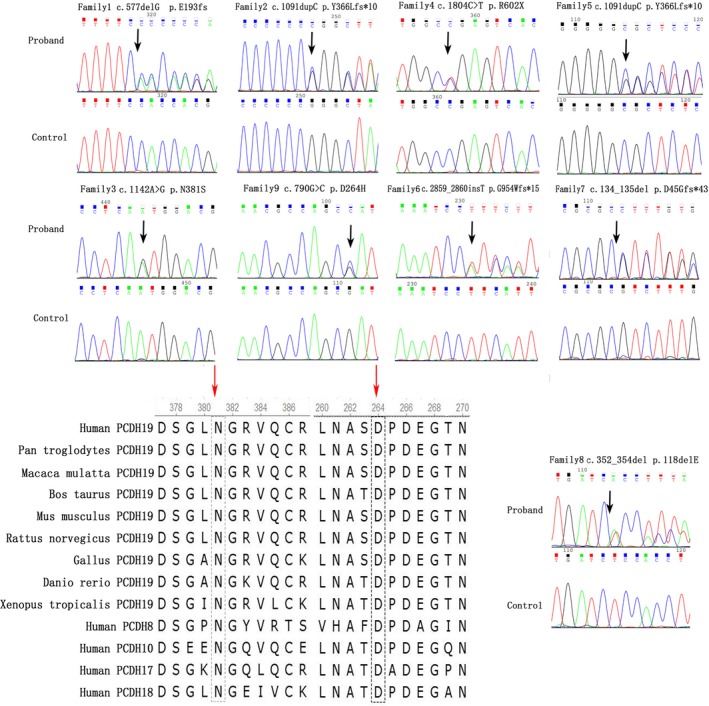
Sequence chromatograms and conversation of amino acid residues affected by the missense mutations. Sequence chromatograms of a *PCDH19* mutation as detected in an affected female is shown for each family. Mutation nomenclature is based on the *PCDH19* transcript reference EF676096. The red arrow upon orthologous and paralogous protein alignments showing the high conservation of each amino acid altered by missense mutations in vertebrates and in the delta 2 protocadherin paralogous genes

**Figure 3:**
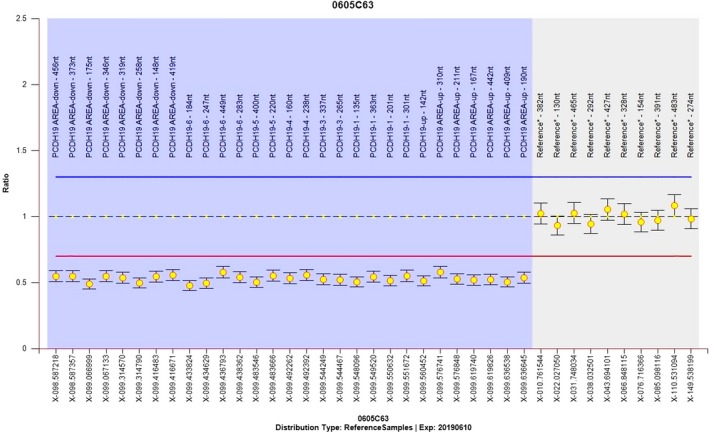
Identification of the *PCDH19* deletion in family 10. *Y*‐axes represent log *R* ratio; the *X*‐axis indicates the position on the X chromosome

**Figure 4:**
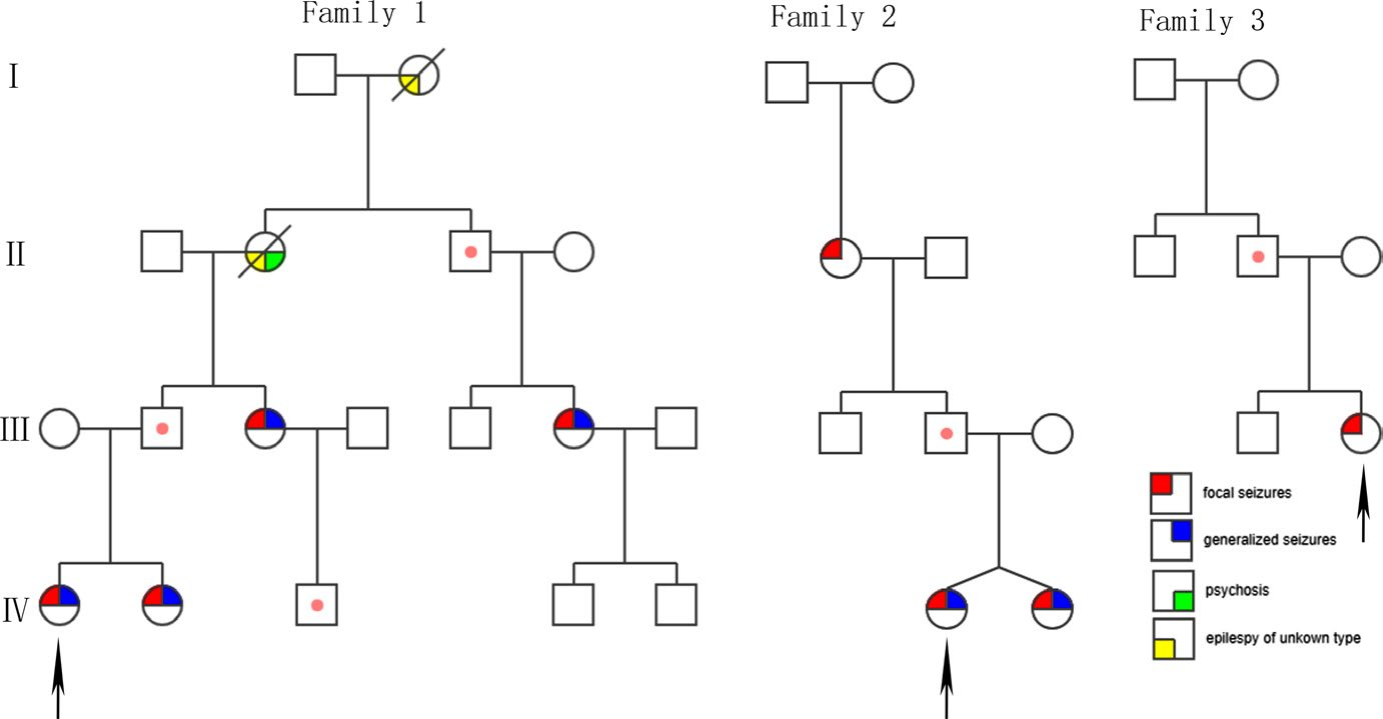
Pedigrees of three females with *PCDH19*‐GCE families. It showing the characteristic inheritance pattern of affected females and transmitting males. Squares represent males, circles females; Upper left corner: focal seizures; Upper right corner: generalized seizures; Lower right corner: psychosis; Lower left corner: Epilepsy of unknown type. Dots in the middle of the squares indicate unaffected mutation carriers. The arrows indicate the proband in the family

We screened 152 children with fever‐sensitive epilepsy for gene detection (85 male and 67 female). We found eight *PCDH19* point mutations and one whole gene deletion, four novel and four reported mutations in 10 female probands who also had cluster seizures (10/152, 6.57%) (Table 1 and Figure 1). Seven mutations were located in exon 1 and one in exon 6. Two missense mutations (c.1142A > G/p.Asn381Ser; c.790G > C/p.Asp264His), one nonsense mutation (c.1804C > T/p.Arg602*), four frameshift mutations, two frameshift deletions (c.577delG/p.Glu193Lysfs*19; c.134_135del/p.Asp45Glyfs*43), two frameshift insertions (c.1091dupC/p.Tyr366Leufs*10;c.2859_2860insT/p.Gly954Trpfs*15), and one in‐frame deletions of three amino acids (c.352_354del/p.Glu118del). Missense mutations all affected amino acids of the extracellular domain of PCDH19, which are highly conserved in orthologs and in paralogs of protocadherins (PCDHs), and were predicted to be pathogenic by MutationTaster, Polyphen2, and SIFT (Figure 2, Table 2). One case was deletion of whole *PCDH19* gene (Figure 3). Seven of the 10 probands for *PCDH19* mutations were de novo (family 4–10). The inheritance of *PCDH19* mutations in three families, females in family No. 1 and No. 2 inherited the mutation from asymptomatic fathers, while the asymptomatic father and the symptomatic aunt inherited from their symptomatic grandmother and great‐grandmother. Family No. 1 was four‐generation pedigrees, family No. 2 was three‐generation pedigrees, and family No. 3 was two‐generation pedigrees (Figure 4).

**Table 1:** Pathogenicity assessment of *PCDH19* mutations

Family	Mutation type	Position: Chr X	Exon	Domain	Amino acid changes	Consequence at the protein level	Parents' analysis	ACMG scoring	ACMG pathogenicity	Reported/Novel
1	Frameshift	100408021	1	EC2	c.577delG	p.Glu193Lysfs*19	Paternal	PVS + PM1 + PM2	P	N
2	Frameshift	99662504–99662505	1	EC4	c.1091dupC	p.Tyr366Leufs*10	Paternal	PVS + PS1 + PM1	P	Y
3	Missense	99662454	1	EC4	c.1142A > G	p.Asn381Ser	Paternal	PM1 + PM2 + PP3 + PP4	LP	N
4	Nonsense	99661792	1	EC6	c.1804C > T	p.Arg602*	De novo	PVS + PS1 + PS2 + PM1 + PM2	P	Y
5	Frameshift	99662504–99662505	1	EC4	c.1091dupC	p.Tyr366Leufs*10	De novo	PVS + PS1 + PS2 + PM1	P	Y
6	Frameshift	99551862	6	CP	c.2859_2860insT	p.Gly954Trpfs*15	De novo	PS2 + PM2	VUS	N
7	Frameshift	99663460–99663462	1	EC1	c.134_135del	p.Asp45Glyfs*43	De novo	PVS + PS1 + PS2 + PM1 + PM2	P	Y
8	In frame	99663242–99663244	1	EC1	c.352_354del	p.Glu118del	De novo	PS2 + PM1 + PM2 + PM4	P	N
9	Missense	99662806	1	EC3	c.790G > C	p.Asp264His	De novo	PS1 + PS2 + PM1 + PM2 + PP3	P	Y
10	Large deletion		1–6		Deletion of exons 1 to 6	Absence of protein synthesis	De novo		P	Y

Abbreviations: CP, cytoplasmic domain; EC, extracellular cadherin domains; LP, likely pathogenic; N, no; P, pathogenic; TM, transmembrane; Y, yes.

**Table 2:** Pathogenicity assessment and conservative analysis of 2 missense mutations

Family	Domain	Amino acid changes	Consequence at the protein level	Parents' analysis	SIFT	Polyphen 2	MutationTaster	GERP++
3	EC4	c.1142A > G	p.Asn381Ser	Paternal	Damaging	Probably damaging	Disease causing	5.95 (Conserved)
9	EC3	c.790G > C	p.Asp264His	De novo	Damaging	Probably damaging	Disease causing	5.95 (Conserved)

Abbreviation: EC, extracellular cadherin domains.

### 3.2 Clinical features

The follow‐up period ranged from 2 months to 4 years. The onset age of 152 children ranged from 6 days after birth to 7 years old. Twenty two patients were diagnosed as DS. One hundred and twenty six cases had generalized tonic‐clonic seizures, 98 had focal seizures, three had spastic seizures, five had myoclonic seizures, one had atypical absence seizures, and one had typical absence seizures. EEG revealed focal spike‐waves in 28 cases, 15 showed generalized spike‐waves, 12 had multiple focal discharges, and two had hypsarrhythmia. Two had enlargement of the subarachnoid space and two had enlarged lateral ventricles in cranial MRI. Forty three cases had family history of epilepsy or febrile convulsion. Thirty six cases were treated with three or more antiepileptic drugs (AEDs). Thirty one with uncontrolled seizures and 48 accompanied with developmental retardation.

**Figure 5:**
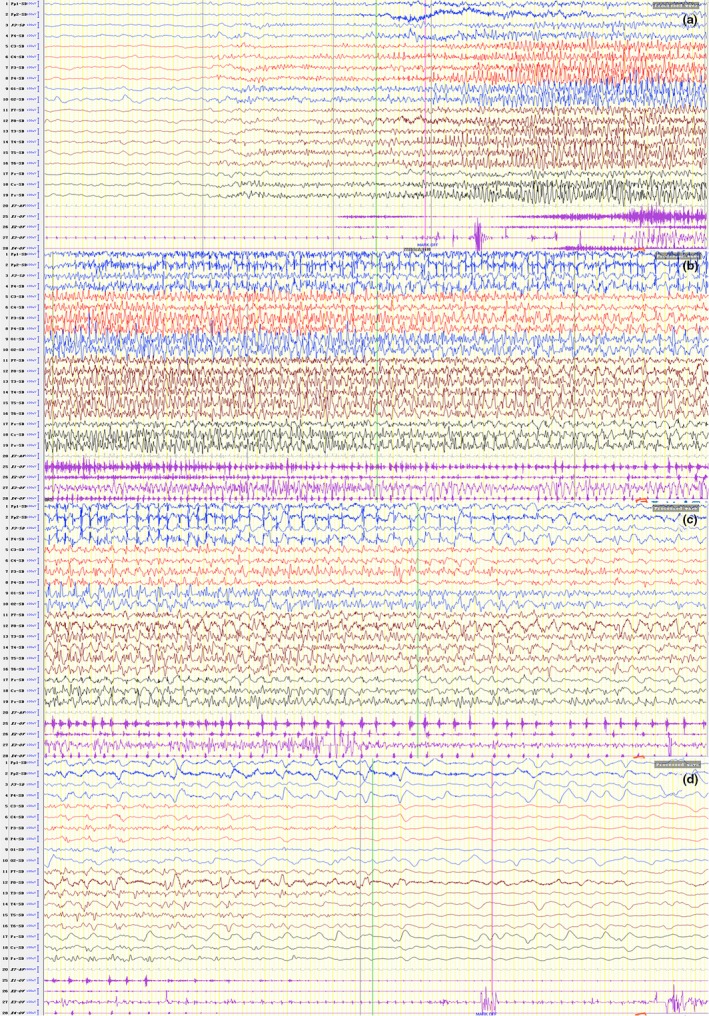
Ictal EEG and EMG tracing of patient No. 10 in family 4. Rapid spikes followed by sharp waves, predominant on the right central‐parietal regions corresponding to rhythmic shaking of left limbs and eyelids

The clinical information of 16 patients with PCDH19 mutations in 10 families is summarized in Table 3. The onset age of the 16 patients ranged from 5 months to 20 years old. The mode of these 16 patients is 3 years old, median is 1.7 years old. The type of seizures was unknown in the deceased grandmother and great‐grandmother of family No. 1. All the other 14 patients had focal motor seizures (14/14, 100%). Five of them had “screaming” at the beginning of seizures (5/14, 35.71%), and five had generalized tonic‐clonic seizures (GTCS) (5/14, 35.71%). Thirteen patients had fever sensitivity (13/14, 93.75%), excepting the grandmother in family No. 2 with onset age of 20 years old. All of them had transient seizures, mostly within one minute, and no status epilepticus occurred. Each cluster with or without fever contained focal motor seizures or GTCS 2–40 times a day, lasting for 1–5 days. Two patients had normal intelligence (2/16, 12.5%), five patients had no language retardation (5/16, 31.25%), and the others had mild‐to‐moderate retardation. The grandmother in family No. 1 had psychosis, including schizophrenia and aggressive behavior (1/16, 6.25%). Fourteen patients had interictal electroencephalogram (EEG) at least one time. Four patients had multiple or focal transient discharges in interictal EEG (4/14, 28.57%), and no epileptic discharges were observed after periodic follow‐up. Focal seizures were captured on EEG in seven patients. Focal seizures of four patients (4/7, 57.14%) originating in the frontal region were recorded, two in the parietal region (Figure 5) (2/7, 28.57%), and one in the occipital region (1/7, 14.29%). Two patients had noncharacteristic anomaly on cranial MRI as lateral ventricular enlargement (2/14, 14.29%).

**Table 3:** The clinical manifestations of the female epileptic patients with *PCDH19* mutations

Family	Patient	Age at exam (years.month)	Onset age (years.month)	Type of seizures	SE	Seizures in cluster	Sensitivity to fever	Intellectual disability	Language delay	Autism	Psychosis	Brain MRI	Interictal EEG	Onset area of focal sz	The last follow‐up		
															Present age	Current AEDs	Seizure frequency
1	1	5.6	2.6	Focal	N	Y	Y	Mild	Mild	N	N	Normal	Normal	Left frontal	7.5	LEV/VPA	Seizure free for 2 years
1	2	2.3	0.6	Focal	N	Y	Y	Moderate	Moderate	N	N	Normal	Normal	Right frontal	4.2	LEV/VPA/TPM	Seizure free for 1 year
1	3	28	8	GTCS Focal	N	Y	Y	Moderate	Moderate	N	N	Normal	Normal	NA	30	N	Seizure free for 13 years
1	4		3	NA	NA	NA	NA	Moderate	Moderate	N	Y	NA	NA	NA		NA	NA
1	5		3	NA	NA	NA	NA	Moderate	Moderate	N	N	NA	NA	NA		NA	NA
2	6	6	1.5	Focal	N	Y	Y	Very mild	N	N	N	Normal	Normal	NA	7	OXC/CZP	Seizure free for 2 years
2	7	6	1.9	Focal	N	Y	Y	Very mild	N	N	N	Normal	Normal	NA	7	OXC/CZP	Seizure free for 2 years
2	8	56	20	Focal	N	Y	N	N	N	N	N	Normal	Normal	NA	57	CZB	Seizure free for 3 years
3	9	2.1	1.1	GTCS Focal	N	Y	Y	N	N	N	N	Normal	Normal	NA	2.3	VPA	1–2 clusters a year
4	10	1	0.5	GTCS Focal	N	Y	Y	Mild	Moderate	N	N	Normal	Normal	Right parietal	2.2	VPA/TPM/CZP	Seizure free for 1 year
5	1	1.11	0.11	Focal	N	Y	Y	Moderate	Moderate	N	N	Normal	FD	Right frontal	3	VPA	Seizure free for 1 year
6	12	2.6	1.4	Focal	N	Y	Y	Moderate	Moderate	N	N	Normal	Normal	Left occipital	3.8	VPA/TPMNZP/OXC	3–4 clusters a year
7	13	5.5	3.4	Focal	N	Y	Y	Very mild	N	N	N	Normal	FD	Left frontal	7.6	VPA	Seizure free for 2 years
8	14	6	0.7	Focal	N	Y	Y	Mild	Mild	N	N	Enlargement of lateral ventricular	FD	NA	9.6	VPA/TPMLTG/OXC	1–2 clusters a year
9	15	0.10	0.8	GTCS Focal	N	Y	Y	Mild	Mild	N	N	Enlargement of ventricular and subarachnoid space in left brain	Multi. FD	Right parietal	2.1	VPA/TPM	1–2 clusters a year
10	16	7.8	1.6	GTCS Focal	N	Y	Y	Moderate/severe	Moderate	N	N	Normal	Normal	NA	9.2	LEV/VPA/TPM	1–2 clusters a year

Abbreviations: AEDs, antiepileptic drugs; CBZ, carbamazepine; CZP, clonazepam; FD, focal discharge; Focal, focal motor seizures; GTCS, generalized tonic‐clonic seizures; LEV, levetiracetam; LTG, lamotrigine; Multi. FD, multifocal discharge; N, none; NA, not available; NZP, nitrazepam; OXC, oxcarbazepine; TPM, topiramate; VPA, sodium valproate; Y, yes.

Except for patient No. 11, seizures were not sensitive to AEDs in patients. The used AEDs included sodium valproate (VPA), carbamazepine (CBZ), topiramate (TPM), levetiracetam (LEV), lamotrigine (LTG), oxcarbazepine (OXC), clonazepam (CZP), and nitrazepam (NZP). Eight patients needed midazolam in the treatment of acute cluster termination. During the recent follow‐up (the present age range from 2 years and 2 months to 57 years old), one patients did not use AEDs because of seizure‐free. Four patients used single‐drug therapy (4/14, 28.57%), four patients used double‐drug therapy (4/14, 28.57%), and five patients used multi‐drug therapy (5/14, 35.71%). The seizure‐free interval ranged from 3 months to 13 years. Family No. 1 and No. 2 had two affected sisters separately. Sisters in family No. 1 were sensitive to LEV combined VPA. Family No. 2 had twin sisters. The proband was not sensitive to LEV and VPA but to OXC and CZP, and her late‐onset sister was benefit from given OXC and VPA directly to control seizures.

## 4 DISCUSSION

*PCDH19*‐GCE is a special X‐linked inheritance, female heterozygotes are affected (Kolc et al., 2019), male hemizygotes are not affected, neither dominant inheritance nor recessive inheritance. “Cellular interference” mechanism is the main hypothesis to explain this particular genetic pattern. This hypothesis speculates that in normal women without *PCDH19* mutation, cells only express wild‐type PCDH19, which ensures that the organism is in a homozygous neural network environment without pathogenicity. When individuals express two different PCDH19 in heterozygous mutation, the normal interaction between cells will be disturbed. Males did not develop the disease because they expressed only one type of PCDH19 (mutant or wild type). The findings of male mosaic patients seem to confirm the hypothesis (Depienne et al., 2009; Terracciano et al., 2016; Thiffault et al., 2016). A total of 16 patients in 10 families were female, and no mosaic male was found. The genetic pattern was consistent with the hypothesis of cell interference mechanism.

*PCDH19*‐GCE is caused by *PCDH19* mutations, and the protein PCDH19 which encoded by *PCDH19* is expressed in various embryonic and human tissues (Cooper et al., 2015), including kidney, lung, and trachea, but it is significantly expressed in the nervous system, especially in limbic system (such as amygdala, hippocampus, and ventral hypothalamus) and cortex (Hertel, Redies, & Medina, 2012; Kim et al., 2010; Pederick et al., 2016; Schaarschuch & Hertel, 2018), and acts on the proliferation of progenitor cells and formation of nerve circuits and regulation of nerve activity (Bassani et al., 2018; Fujitani, Zhang, Fujiki, Fujihara, & Yamashita, 2017; Hayashi et al., 2017). In neurons, this regulation is the basis for the transmission and integration of synaptic inputs, and establishes adequate responses for the development, plasticity, and survival of neurons (Compagnucci et al., 2015; Kurian et al., 2018; Pederick et al., 2018). PCDH19 belongs to δ2 subgroup of nonclustered desmosomal cadherins and PCDHs, which also contain PCDH8, PCDH10, PCDH12, PCDH17, and PCDH18. This is also the basis for our conservative analysis. *PCDH19* consists of six exons with a total length of 9,765 nucleotides and is located on chromosome Xq22.3. PCDH19 which with 1,148 amino acids is composed of a signal sequence, six extracellular cadherin (EC) repeats, a transmembrane domain and a cytoplasmic region with conserved cytoplasmic domain (CM) 1 and CM2. Exon 1 is translated into the whole extracellular and transmembrane domain, as well as a small part of the cytoplasmic domain. The rest is encoded by exon 2–6. Exons 5 and 6 encode CM1 and CM2, respectively. The reported mutation types include missense, nonsense, insertion or deletion of bases, splicing, deletion of fragments or whole genes, and deletion of other adjacent genes (Depienne et al., 2011; Vincent et al., 2012). Seven of the eight‐point mutations in our study were in exon 1, including four frameshift mutations, two missense mutations, one in‐frame mutation; while one mutation occurred in exon 6, just like the reported mutations which occurred mostly in exon 1 (Kolc et al., 2019). The intracellular C terminal tail of PCDH19 contains the conserved motifs CM1 and CM2, and the Wiskott–Aldrich syndrome protein (WASP) family (Chen et al., 2014), these domains anchor on the cytoskeleton and integrate with intracellular signal transduction pathways. So C.2859_2860insT in exon 6 may play a pathogenic role by affecting intracellular signal transduction. But there is no intron after the premature termination codon this variant generates so the *PCDH19* mRNA is unlikely to be degraded by nonsense‐mediated mRNA decay, and we did not have enough evidences to prove that this variant affects mRNA or protein stability. So according to the ACMG standard, it should be likely pathogenic (LP), but because of its location, it may be just considered as variant of undetermined significance (VUS). The girl with this mutation had fever sensitivity, clustered transient partial seizures, and developmental retardation, the clinical characterization was highly consistent with *PCDH19*‐GCE. So, we kept this VUS site which may be a pathogenic mutation. With the increasing reports of this kind of variants in the future may help us better understand the pathogenesis of *PCDH19*‐GCE. Some evidences proved the cytoplasmic region of PCDH19 which bind to GABA_A_ receptor (GABA_A_R) alpha subunit can regulate the availability of receptor surface which may be involved in the regulation of intracellular transport of GABA_A_R (Bassani et al., 2018; Gerosa et al., 2019). Other studies have found that the level of allopregnanolone (AP) in *PCDH19* female epilepsy patients was decreased (Tan et al., 2015), and AP is the most effective positive regulator of GABA_A_R which mediates fast inhibitory neurotransmission in the brain, so *PCDH19* as a key modulator of GABAergic transmission and may suggest new pathogenic mechanisms. Six mutations were found in EC1–4 (6/8, 75%), including two missense mutations, which are highly conserved in orthologs and in paralogs of PCDHs. The EC1–4 repeats of PCDH19 have been identified as the minimal adhesive unit involved in the generation of a trans adhesive interface. These repeats interact in antiparallel PCDH19 dimer (forearm handshake model) to play the role of adhesion and mediation (Cooper, Jontes, & Sotomayor, 2016).

In this study, Seven *PCDH19* mutations of the 10 probands were de novo, three mutations were inherited from fathers, and sporadic new mutations accounted for the majority, which was the main mutation type, consistent with the literature (Duszyc et al., 2015). And, our report found four novel mutations which expand the spectrum of *PCDH19* mutations associated with epilepsy in females. All the patients in the study were female, with onset age ranging from 5 months to 20 years old, later than the 6 months to 3 years old reported in the literature (Smith et al., 2018). In our study, the patients with the onset age older than 3 years (5/16, 31.25%) were mainly the first and second generations of the families. So *PCDH19*‐GCE does not only onset in infancy, but it also can occur late in adulthood, and the late‐onset patients may have mild clinical phenotype. Except for two deaths, other patients had the characteristics of cluster, transient focal seizures, or GTCS. It was found that 70.5% of children had terrible screams when focal seizures occurred (Antelmi et al., 2012; Marini et al., 2012), the proportion in this study was 35.71%, which was lower than that. Except for one case, all patients had the characteristics of fever sensitivity (15/16, 93.75%), and no status epilepticus in all the patients which was the biggest difference between *PCDH19*‐GCE and DS. And, the onset age of DS was <1 year old, mostly 5–8 months, the ratio of male to female is 2:1, photosensitivity, myoclonic seizures are more common, EEG is normal within 1 year old, and worse gradually in later stages, and most of the cranial MRI was normal. A few DS with hippocampal sclerosis had severe cognitive impairment. 70%‐80% of the children had *SCN1A* mutations and poor prognosis (Trivisano et al., 2016). It is difficult to distinguish *PCDH19*‐GCE from DS, especially in the early stages of onset. Molecular identification of potential genetic defects in epilepsy and analysis of parental status is essential to provide appropriate genetic counseling for families.

Nearly half of the patients had normal intelligence or mild mental retardation, and the remaining half of the group had moderate mental retardation. One patient had psychosis, van Harssel et al. (2013) reported four male carriers of *PCDH19* mutations without epileptic seizures, but with emotional disorders (schizophrenia and autism) and mental retardation. It can be seen that patients with *PCDH19*‐GCE may have normal intelligence and language, mild‐to‐moderate mental retardation, psychotic disorders. Some studies have found schizophrenia and other psychotic disorders as a later‐onset manifestation of *PCDH19*‐GCE (Vlaskamp et al., 2019). We will continue to follow up these patients to see if they manifested mental disorders in the future. There were only two cases that had nonspecific abnormalities on cranial MRI. Although some studies found cortical dysplasia in *PCDH19* mutation patients and mouse models (Kurian et al., 2018; Pederick et al., 2018), this was not found in our patients. Interictal EEG showed transient focal or multiple epileptiform discharges in only four patients. Frontal‐parietal regions as the onset of focal seizures were mainly involved in the ictal EEG. And frontal‐parietal region is predominantly responsible for motion, this may explain focal motor seizures as the main types of focal seizures in *PCDH19*‐GCE.

Children with *PCDH19* mutations often show significant cluster seizures in the early stage of onset, and the effect of AEDs is poor. At present, it has been found that carbamazepine, lamotrigine, and aminohexenoic acid ineffective or aggravated. Sodium valproate, clobazam, phenytoin, and stiripentol are relatively effective (Higurashi et al., 2013; Lotte et al., 2016; Trivisano, Specchio, & Vigevano, 2015). Corticosteroids have been reported to be effective in the treatment of acute cluster termination in a patient with *PCDH19*‐GCE, then the hypothesis that blood–brain barrier dysfunction exists in *PCDH19*‐GCE patients has been proposed (Bertani et al., 2015; Higurashi et al., 2015). However, remission is only temporary and epileptic seizures recur. After discovering that the level of allopregnanolone and steroidogenesis decreased (Tan et al., 2015; Trivisano et al., 2017), clinical trials began with ganaxolone, a synthetic analog of isoprogesterone (Tan et al., 2015). Surgical excision can reduce seizures in patients with cortical dysplasia (Kurian et al., 2018). Children with *PCDH19*‐GCE had better responsiveness to benzodiazepines in the acute stage, and midazolam could control epileptic seizures. Eight patients needed midazolam to help control the acute cluster seizures in the study; however, soon after midazolam was reduced or discontinued, the seizures resurged. Considering the unique pattern of epilepsy, even in the case of continuous seizures, added multiple AEDs quickly should be avoided. In this group of patients, except for patient No. 12, seizures were not sensitive to AEDs. Family No. 1 and No. 2 had two affected sisters separately. The sisters in the same family have the same reactivity to AEDs. This may suggest that if we met a *PCDH19* family in clinic, the medication characteristics of proband in the family can guide the treatment of other patients in the same family. But the number of samples is too small, so that is just our guess. During the recent follow‐up, the seizure‐free interval ranged from 3 months to 13 years. Two patients had remission time longer than 3 years, with remission ages of adolescence and adulthood, respectively. Specchio et al. (2011) believed that AEDs could not control the cluster seizures in children, and the decrease in seizures with age may be related to the decrease in febrile diseases.

In conclusion, *PCDH19* mutations can be inherited or de novo. *PCDH19* mutations‐related epilepsy has incomplete penetration rate and phenotypic heterogeneity. The phenotypes of *PCDH19* mutations include *PCDH19*‐GCE with or without mental retardation, psychosis, and male asymptomatic carriers. *PCDH19*‐GCE is characterized by clustered transient GTCS and focal seizures, and fever sensitivity. The onset age of *PCDH19*‐GCE can vary from childhood to adulthood. And, our report expands the spectrum of *PCDH19* mutations associated with epilepsy in females.

## CONFLICT OF INTEREST

All authors declare that there is no conflict of interest.

## Data Availability

The data that support the findings of this study are available from the corresponding author upon reasonable request.

## References

[brb31455-bib-0001] Antelmi, E. , Mastrangelo, M. , Bisulli, F. , Spaccini, L. , Stipa, C. , Mostacci, B. , … Tinuper, P. (2012). Semiological study of ictal affective behaviour in epilepsy and mental retardation limited to females (EFMR). Epileptic Disorders, 14(3), 304–309. 10.1684/epd.2012.0526 22932693

[brb31455-bib-0002] Bassani, S. , Cwetsch, A. W. , Gerosa, L. , Serratto, G. M. , Folci, A. , Hall, I. F. , … Passafaro, M. (2018). The female epilepsy protein PCDH19 is a new GABAAR‐binding partner that regulates GABAergic transmission as well as migration and morphological maturation of hippocampal neurons. Human Molecular Genetics, 27(6), 1027–1038. 10.1093/hmg/ddy019 29360992 PMC5886308

[brb31455-bib-0003] Bertani, G. , Spagnoli, C. , Iodice, A. , Salerno, G. G. , Frattini, D. , & Fusco, C. (2015). Steroids efficacy in the acute management of seizure clusters in one case of PCDH19 female epilepsy. Seizure, 32, 45–46. 10.1016/j.seizure.2015.09.002 26552561

[brb31455-bib-0004] Chen, B. , Brinkmann, K. , Chen, Z. , Pak, C. W. , Liao, Y. , Shi, S. , … & Rosen, M. K. (2014). The WAVE regulatory complex links diverse receptors to the actin cytoskeleton. Cell, 156(1–2), 195–207. 24439376 10.1016/j.cell.2013.11.048 PMC4059610

[brb31455-bib-0005] Compagnucci, C. , Petrini, S. , Higuraschi, N. , Trivisano, M. , Specchio, N. , Hirose, S. , … Terracciano, A. (2015). Characterizing PCDH19 in human induced pluripotent stem cells (iPSCs) and iPSC‐derived developing neurons: Emerging role of a protein involved in controlling polarity during neurogenesis. Oncotarget, 6(29), 26804–26813. 10.18632/oncotarget.5757 26450854 PMC4694954

[brb31455-bib-0006] Cooper, S. R. , Emond, M. R. , Duy, P. Q. , Liebau, B. G. , Wolman, M. A. , & Jontes, J. D. (2015). Protocadherins control the modular assembly of neuronal columns in the zebrafish optic tectum. Journal of Cell Biology, 211(4), 807–814. 10.1083/jcb.201507108 26598617 PMC4657173

[brb31455-bib-0007] Cooper, S. R. , Jontes, J. D. , & Sotomayor, M. (2016). Structural determinants of adhesion by Protocadherin‐19 and implications for its role in epilepsy. eLife, 5, 10.7554/eLife.18529 PMC5115871 27787195

[brb31455-bib-0008] Depienne, C. , Bouteiller, D. , Keren, B. , Cheuret, E. , Poirier, K. , Trouillard, O. , … LeGuern, E. (2009). Sporadic infantile epileptic encephalopathy caused by mutations in PCDH19 resembles Dravet syndrome but mainly affects females. PLoS Genetics, 5(2), e1000381 10.1371/journal.pgen.1000381 19214208 PMC2633044

[brb31455-bib-0009] Depienne, C. , & LeGuern, E. (2012). PCDH19‐related infantile epileptic encephalopathy: An unusual X‐linked inheritance disorder. Human Mutation, 33(4), 627–634. 10.1002/humu.22029 22267240

[brb31455-bib-0010] Depienne, C. , Trouillard, O. , Bouteiller, D. , Gourfinkel‐An, I. , Poirier, K. , Rivier, F. , … LeGuern, E. (2011). Mutations and deletions in PCDH19 account for various familial or isolated epilepsies in females. Human Mutation, 32(1), E1959–E1975. 10.1002/humu.21373 21053371 PMC3033517

[brb31455-bib-0011] Duszyc, K. , Terczynska, I. , & Hoffman‐Zacharska, D. (2015). Epilepsy and mental retardation restricted to females: X‐linked epileptic infantile encephalopathy of unusual inheritance. Journal of Applied Genetics, 56(1), 49–56. 10.1007/s13353-014-0243-8 25204757

[brb31455-bib-0012] Fujitani, M. , Zhang, S. , Fujiki, R. , Fujihara, Y. , & Yamashita, T. (2017). A chromosome 16p13.11 microduplication causes hyperactivity through dysregulation of miR‐484/protocadherin‐19 signaling. Molecular Psychiatry, 22(3), 364–374. 10.1038/mp.2016.106 27378146 PMC5322274

[brb31455-bib-0013] Gerosa, L. , Francolini, M. , Bassani, S. , & Passafaro, M. (2019). The role of protocadherin 19 (PCDH19) in neurodevelopment and in the pathophysiology of early infantile epileptic encephalopathy‐9 (EIEE9). Developmental Neurobiology, 79(1), 75–84. 10.1002/dneu.22654 30431232

[brb31455-bib-0014] Hayashi, S. , Inoue, Y. , Hattori, S. , Kaneko, M. , Shioi, G. , Miyakawa, T. , & Takeichi, M. (2017). Loss of X‐linked Protocadherin‐19 differentially affects the behavior of heterozygous female and hemizygous male mice. Scientific Reports, 7(1), 5801 10.1038/s41598-017-06374-x 28724954 PMC5517645

[brb31455-bib-0015] Hertel, N. , Redies, C. , & Medina, L. (2012). Cadherin expression delineates the divisions of the postnatal and adult mouse amygdala. Journal of Comparative Neurology, 520(17), 3982–4012. 10.1002/cne.23140 22592879

[brb31455-bib-0016] Higurashi, N. , Nakamura, M. , Sugai, M. , Ohfu, M. , Sakauchi, M. , Sugawara, Y. , … Hirose, S. (2013). PCDH19‐related female‐limited epilepsy: Further details regarding early clinical features and therapeutic efficacy. Epilepsy Research, 106(1–2), 191–199. 10.1016/j.eplepsyres.2013.04.005 23712037

[brb31455-bib-0017] Higurashi, N. , Takahashi, Y. , Kashimada, A. , Sugawara, Y. , Sakuma, H. , Tomonoh, Y. , … Hirose, S. (2015). Immediate suppression of seizure clusters by corticosteroids in PCDH19 female epilepsy. Seizure, 27, 1–5. 10.1016/j.seizure.2015.02.006 25891919

[brb31455-bib-0018] Homan, C. C. , Pederson, S. , To, T.‐H. , Tan, C. , Piltz, S. , Corbett, M. A. , … Gecz, J. (2018). PCDH19 regulation of neural progenitor cell differentiation suggests asynchrony of neurogenesis as a mechanism contributing to PCDH19 Girls Clustering Epilepsy. Neurobiology of Diseases, 116, 106–119. 10.1016/j.nbd.2018.05.004 29763708

[brb31455-bib-0019] Kim, S. Y. , Mo, J. W. , Han, S. , Choi, S. Y. , Han, S. B. , Moon, B. H. , … Kim, H. (2010). The expression of non‐clustered protocadherins in adult rat hippocampal formation and the connecting brain regions. Neuroscience, 170(1), 189–199. 10.1016/j.neuroscience.2010.05.027 20541594

[brb31455-bib-0020] Kolc, K. L. , Sadleir, L. G. , Scheffer, I. E. , Ivancevic, A. , Roberts, R. , Pham, D. H. , & Gecz, J. (2019). A systematic review and meta‐analysis of 271 PCDH19‐variant individuals identifies psychiatric comorbidities, and association of seizure onset and disease severity. Molecular Psychiatry, 24(2), 241–251. 10.1038/s41380-018-0066-9 29892053 PMC6344372

[brb31455-bib-0021] Kurian, M. , Korff, C. M. , Ranza, E. , Bernasconi, A. , Lubbig, A. , Nangia, S. , … Bast, T. (2018). Focal cortical malformations in children with early infantile epilepsy and PCDH19 mutations: Case report. Developmental Medicine and Child Neurology, 60(1), 100–105. 10.1111/dmcn.13595 29064093

[brb31455-bib-0022] Leonardi, E. , Sartori, S. , Vecchi, M. , Bettella, E. , Polli, R. , Palma, L. D. , … Murgia, A. (2014). Identification of four novel PCDH19 Mutations and prediction of their functional impact. Annals of Human Genetics, 78(6), 389–398. 10.1111/ahg.12082 25227595

[brb31455-bib-0023] Lotte, J. , Bast, T. , Borusiak, P. , Coppola, A. , Cross, J. H. , Dimova, P. , … Kluger, G. (2016). Effectiveness of antiepileptic therapy in patients with PCDH19 mutations. Seizure, 35, 106–110. 10.1016/j.seizure.2016.01.006 26820223

[brb31455-bib-0024] Marini, C. , Darra, F. , Specchio, N. , Mei, D. , Terracciano, A. , Parmeggiani, L. , … Guerrini, R. (2012). Focal seizures with affective symptoms are a major feature of PCDH19 gene‐related epilepsy. Epilepsia, 53(12), 2111–2119. 10.1111/j.1528-1167.2012.03649.x 22946748

[brb31455-bib-0025] Pederick, D. T. , Homan, C. C. , Jaehne, E. J. , Piltz, S. G. , Haines, B. P. , Baune, B. T. , … Thomas, P. Q. (2016). Pcdh19 loss‐of‐function increases neuronal migration in vitro but is dispensable for brain development in mice. Scientific Reports, 6, 26765 10.1038/srep26765 27240640 PMC4886214

[brb31455-bib-0026] Pederick, D. T. , Richards, K. L. , Piltz, S. G. , Kumar, R. , Mincheva‐Tasheva, S. , Mandelstam, S. A. , … Thomas, P. Q. (2018). Abnormal cell sorting underlies the unique X‐linked inheritance of PCDH19 epilepsy. Neuron, 97(1), 59–66.e55. 10.1016/j.neuron.2017.12.005 29301106

[brb31455-bib-0027] Schaarschuch, A. , & Hertel, N. (2018). Expression profile of N‐cadherin and protocadherin‐19 in postnatal mouse limbic structures. Journal of Comparative Neurology, 526(4), 663–680. 10.1002/cne.24359 29159962

[brb31455-bib-0028] Smith, L. , Singhal, N. , El Achkar, C. M. , Truglio, G. , Rosen Sheidley, B. , Sullivan, J. , & Poduri, A. (2018). PCDH19‐related epilepsy is associated with a broad neurodevelopmental spectrum. Epilepsia, 59(3), 679–689. 10.1111/epi.14003 29377098 PMC6264912

[brb31455-bib-0029] Specchio, N. , Marini, C. , Terracciano, A. , Mei, D. , Trivisano, M. , Sicca, F. , … Vigevano, F. (2011). Spectrum of phenotypes in female patients with epilepsy due to protocadherin 19 mutations. Epilepsia, 52(7), 1251–1257. 10.1111/j.1528-1167.2011.03063.x 21480887

[brb31455-bib-0030] Tan, C. , Shard, C. , Ranieri, E. , Hynes, K. , Pham, D. H. , Leach, D. , … Gecz, J. (2015). Mutations of protocadherin 19 in female epilepsy (PCDH19‐FE) lead to allopregnanolone deficiency. Human Molecular Genetics, 24(18), 5250–5259. 10.1093/hmg/ddv245 26123493

[brb31455-bib-0031] Terracciano, A. , Trivisano, M. , Cusmai, R. , De Palma, L. , Fusco, L. , Compagnucci, C. , … Specchio, N. (2016). PCDH19‐related epilepsy in two mosaic male patients. Epilepsia, 57(3), e51–55. 10.1111/epi.13295 26765483

[brb31455-bib-0032] Thiffault, I. , Farrow, E. , Smith, L. , Lowry, J. , Zellmer, L. , Black, B. , … Saunders, C. (2016). PCDH19‐related epileptic encephalopathy in a male mosaic for a truncating variant. American Journal of Medical Genetics. Part A, 170(6), 1585–1589. 10.1002/ajmg.a.37617 27016041

[brb31455-bib-0033] Trivisano, M. , Lucchi, C. , Rustichelli, C. , Terracciano, A. , Cusmai, R. , Ubertini, G. M. , … Specchio, N. (2017). Reduced steroidogenesis in patients with PCDH19‐female limited epilepsy. Epilepsia, 58(6), e91–e95. 10.1111/epi.13772 28471529

[brb31455-bib-0034] Trivisano, M. , Pietrafusa, N. , Ciommo, V. D. , Cappelletti, S. , Palma, L. D. , Terracciano, A. , … Specchio, N. (2016). PCDH19‐related epilepsy and Dravet syndrome: Face‐off between two early‐onset epilepsies with fever sensitivity. Epilepsy Research, 125, 32–36. 10.1016/j.eplepsyres.2016.05.015 27371789

[brb31455-bib-0035] Trivisano, M. , Specchio, N. , & Vigevano, F. (2015). Extending the use of stiripentol to other epileptic syndromes: A case of PCDH19‐related epilepsy. European Journal of Paediatric Neurology, 19(2), 248–250. 10.1016/j.ejpn.2014.11.008 25510386

[brb31455-bib-0036] van Harssel, J. J. T. , Weckhuysen, S. , van Kempen, M. J. A. , Hardies, K. , Verbeek, N. E. , de Kovel, C. G. F. , … Brilstra, E. H. (2013). Clinical and genetic aspects of PCDH19‐related epilepsy syndromes and the possible role of PCDH19 mutations in males with autism spectrum disorders. Neurogenetics, 14(1), 23–34. 10.1007/s10048-013-0353-1 23334464

[brb31455-bib-0037] Vincent, A. K. , Noor, A. , Janson, A. , Minassian, B. A. , Ayub, M. , Vincent, J. B. , & Morel, C. F. (2012). Identification of genomic deletions spanning the PCDH19 gene in two unrelated girls with intellectual disability and seizures. Clinical Genetics, 82(6), 540–545. 10.1111/j.1399-0004.2011.01812.x 22091964

[brb31455-bib-0038] Vlaskamp, D. R. M. , Bassett, A. S. , Sullivan, J. E. , Robblee, J. , Sadleir, L. G. , Scheffer, I. E. , & Andrade, D. M. (2019). Schizophrenia is a later‐onset feature of PCDH19 Girls Clustering Epilepsy. Epilepsia, 60(3), 429–440. 10.1111/epi.14678 30828795

